# The Seasonal Spatial Distribution Pattern and Migration of Kishi Velvet Shrimp *Metapenaeopsis dalei* in the Southern Yellow and East China Seas

**DOI:** 10.3390/ani16020296

**Published:** 2026-01-18

**Authors:** Min Xu, Xiaojing Song, Yang Xu, Jianzhong Ling, Huiyu Li

**Affiliations:** Key Laboratory of Fisheries Remote Sensing Ministry of Agriculture and Rural Affairs, East China Sea Fisheries Research Institute, Chinese Academy of Fishery Sciences, Shanghai 200090, China; xuminwzy@aliyun.com (M.X.); songxiaojing@ecsf.ac.cn (X.S.); yolanda202510@126.com (Y.X.)

**Keywords:** decapoda, East Asia, East China Seas, northern China, Northwestern Pacific, Penaeidae, stock assessment, trawling

## Abstract

It is important to understand the ecological information of *Metapenaeopsis dalei* to better conserve and manage the stocks in Asia. In this study, we employed research vessels to collect the field data including biomass and number of *M. dalei* in each survey stations along with environmental data including depth, water temperature, and salinity from November 2018 to September 2019 in the region of 26.50–35.00° N and 120.00–127.00° E in the southern Yellow and East China Seas of China. We found that the annual mean catch per unit effort of weight and number (CPUE_w_ and CPUE_n_) was 15,235.89 g∙h^−1^ and 17,319.13 ind∙h^−1^, respectively. *Metapenaeopsis dalei* was found in 10–130 m. The greatest biomass occurred at 10–20 m in spring, 30–40 m in summer, 10–100 m in autumn, and 10–40 m in winter. The greatest abundance occurred at sea bottom temperature (SBT) 14–15 °C in spring, 19 °C in summer, 15–20 °C in autumn, and 10–12 °C in winter. The greatest abundance occurred at sea bottom salinity (SBS) 32–33 in spring, 32 in summer, 32–35 in autumn, and 31–32 in winter. We found the lowest SBT of *M. dalei* at 10–11 °C in spring and summer. The juveniles were found at SBT 21 °C and SBS 34 in autumn. The total CPUE_w_ and CPUE_n_ rankings were winter > spring > autumn > summer, and the mean average individual weight (AIW) ranking was summer > spring > winter > autumn. Fishing grounds of Haizhou Bay–Lvsi and Zhoushan–Yushan may be the spawning grounds for *M. dalei*. These findings can benefit fishery management action and planning in the future.

## Simple Summary

*Metapenaeopsis dalei* is an economically important shrimp species in Northeast Asia. However, very little ecological information is available about the seasonal spatial distribution and migration pattern of *M. dalei* in the Yellow and East China Seas of China. In this study, we assume that the parent cohorts spawned in the coastal waters of Haizhou Bay–Lvsi in the southern Yellow Sea and Zhoushan–Yushan in the East China Sea in May to August and the newborn recruitment cohorts migrate to adjacent offshore water areas for further nursery and overwintering in other seasons. The diluted water mass from the Yangtze River might play an important role in the spawning and nursery of *M. dalei*. The above findings can benefit the stock conservation and fisheries management of *M. dalei* in China.

## 1. Introduction

In East Asia, including Japan, Korea, and northern China, Kishi velvet shrimp *Metapenaeopsis dalei* (Rathbun 1902) (Malacostraca, Decapoda, Penaeidae) are widely distributed along the brackish and coastal waters [1]. In Japan, *M. dalei* is distributed from the southern waters, including Tosa Bay (33° N), to the northern waters, including Hakodate Bay (41° N), indicating good adaptation to a wide range of water temperatures [2,3]. In Korea, this commercially important shrimp species is endemic to southern and western coastal waters, with its natural distribution extending from Kanghwado Island in the northwest all the way to Goejedo Island in the southeast [4]. The carapace length of *M. dalei* is 40 to 70 mm, and the wet weight is 0.8 to 3.5 g [5]. Growth curves demonstrate that female individuals exhibit a faster growth rate than males, with the smallest sexually mature female specimen measuring 11.0 mm in carapace length [6].

*Metapenaeopsis dalei* is often found in sandy, muddy substrates at 10–130 m depth [1]. They are very often found together with the shrimp *Trachypenaeus curvirostris* [5]. A clear disparity in lifespan was observed between the sexes of *M. dalei*: females live for 15 to 16 months, while males only reach 14 to 15 months of age [6]. The fecundity of *M. dalei* is directly proportional to the size of the female. In the Seto Inland Sea of Japan, the annual production of *M. dalei* was recorded as 20,000 tons, from small-scale bottom trawling [7]. From May to July, *M. dalei* production by the Zhejiang Zhoushan seafood company was 1000 tons in China [5]. Furthermore, in Korean waters, the seasonal rise in water temperatures, commencing in April, triggers the migration of parental M. dalei cohorts from deep offshore waters to inshore coastal regions; consequently, this species is commercially harvested via shrimp trawls and stow nets from April to October [1].

However, very little ecological information is known about the seasonal spatial distribution and migration pattern of *M. dalei* in China. In this study, we aim to identify the seasonal variations of environmental factors (including water temperature, salinity, and depth) and biological parameters (including total number and biomass at each station) of *M. dalei* and further understand the migration and distribution pattern related to environmental factors in the southern Yellow and East China Seas. Our findings are pivotal for the exploitation, conservation, and fisheries management of *M. dalei* in China.

## 2. Materials and Methods

**Figure 1:**
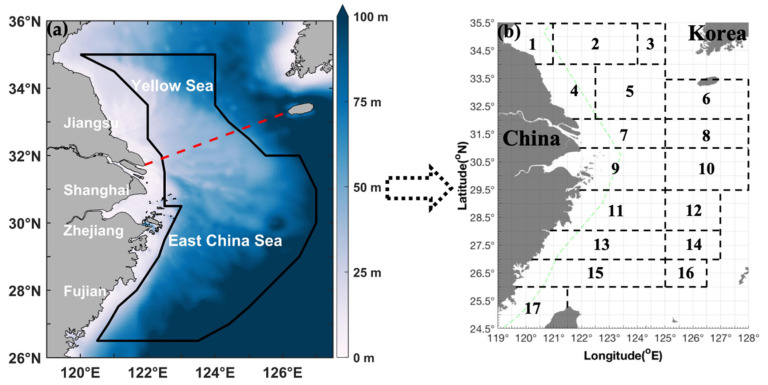
(**a**) Map of the study area (26.50–35.50° N, 119.00–128.00° E): the study area, which encompasses the southern Yellow Sea and East China Sea, is outlined by a red dashed line within the broader East China Sea domain. (**b**) Black rectangular markers and numerical labels correspond to the following fishing grounds: (1) Haizhou Bay, (2) Lianqingshi, (3) Liandong, (4) Lvsi, (5) Dasha, (6) Shawai, (7) Yangtze River Estuary, (8) Jiangwai, (9) Zhoushan, (10) Zhouwai, (11) Yushan, (12) Yuwai, (13) Wentai, (14) Wenwai, (15) Mindong, (16) Minwai, and (17) Minzhong. The green dashed line indicates the motor trawling prohibition boundary.

A series of independent scientific bottom trawling surveys were implemented in 2018–2019 across the southern Yellow Sea and East China Sea, with fieldwork conducted in four discrete seasonal windows: autumn (11 November 2018), winter (27 January 2019), spring (22 April–10 May 2019), and summer (13 August–27 September 2019) (Figure 1). The fishing gear deployed was a standardized trawl net characterized by a 20-mm cod-end mesh, 72.24-m headline length, 10–15-m net height, and 82.44-m groundline length. Trawling was undertaken by fisheries research vessels Zhongkeyu 211 and 212 within the coordinates 26.50–35.00° N and 120.00–127.00° E. Survey stations were allocated based on a 30-min latitude × 30-min longitude grid. Each trawl was performed at a uniform speed of 3 knots and lasted for 1 h per station. The final dataset comprised 519 valid trawling events, distributed as follows: 127 in autumn, 111 in winter, 141 in spring, and 140 in summer.

Following trawling operations, all catches were processed in the laboratory to confirm species identity and document their occurrence at each sampling station. For each station, the complete sample set was counted, and the wet weight was determined with a precision of 0.10 g. Two catch density indices were computed for the focal species: biomass density (CPUE_w_, g h^−1^) and numerical density (CPUE_n_, ind h^−1^), both standardized by sampling time. Average individual weight (AIW) was then calculated as the quotient of CPUE_w_ and CPUE_n_ for each station. A conductivity–temperature–depth (CTD) profiler (SBE-19, Sea-Bird Scientific, Bellevue, WA, USA) was deployed to collect environmental data at every station. Sea surface salinity (SSS) and sea surface temperature (SST) were measured at 3 m below the surface, while bottom water parameters (SBS, SBT) were sampled at variable heights above the seabed: 2 m for water depths <50 m, and 2–4 m for depths exceeding 50 m.

## 3. Results

### 3.1. Seasonal Variations in Depth, Water Temperature, and Salinity

**Figure 2:**
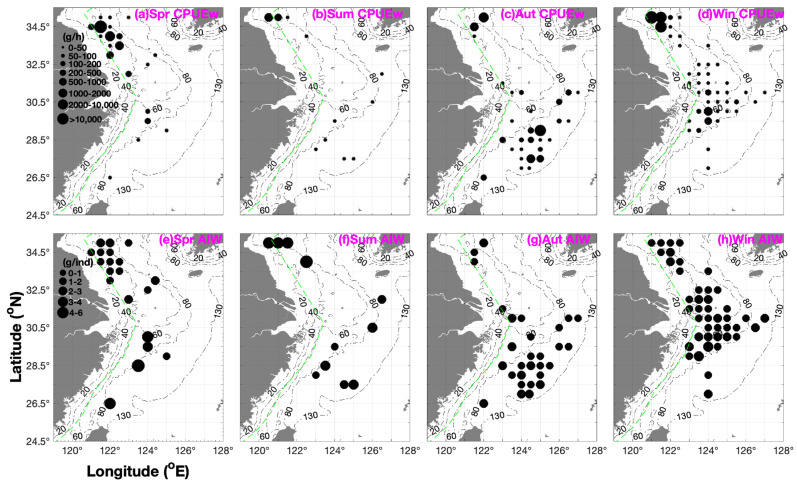
Seasonal spatial distribution patterns of *Metapenaeopsis dalei* catch per unit effort by weight (CPUE_w_, g h^−1^) and average individual weight (AIW, g ind^−1^). CPUE_w_ values are categorized into eight intervals (0–50, 50–100, 100–200, 200–500, 500–1000, 1000–2000, 2000–10000, and >10,000 g h^−1^) and depicted in black; AIW values are grouped into five intervals (0–1, 1–2, 2–3, 3–4, and 4–6 g ind^−1^) and also presented in black. Panels (**a**–**d**) represent CPUE_w_ distributions in spring, summer, autumn, and winter, respectively; panels (**e**–**h**) denote AIW distributions across the same four seasons.

**Figure 3:**
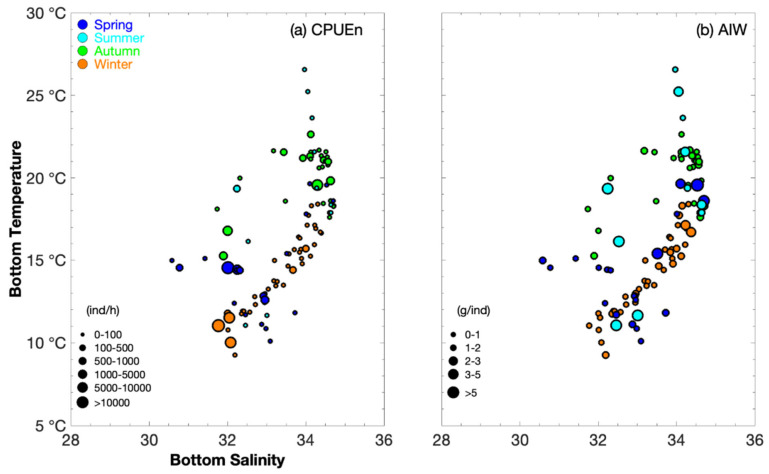
Relationships between sea bottom salinity and sea bottom temperature (°C) with two key catch metrics of *Metapenaeopsis dalei*: numerical catch per unit effort (CPUE_n_) and average individual weight (AIW). CPUE_n_ values were categorized into six intervals (0–100, 100–500, 500–1000, 1000–5000, 5000–10000, and > 10,000 ind h^−1^), while AIW values were grouped into five intervals (0–1, 1–2, 2–3, 3–5, and >5 g ind^−1^). Seasonal data points are represented by distinct symbols: solid blue circles (spring), light blue circles (summer), green circles (autumn), and brown circles (winter), respectively. Panel (**a**) illustrates the relationship between sea bottom temperature and sea bottom salinity for CPUEn; Panel (**b**) depicts the same environmental relationship for AIW.

*Metapenaeopsis dalei* was distributed at a depth range of 10–130 m across the season (Table 1). The greatest biomass occurred at depth 10–20 m in spring, 30–40 m in summer, 10–100 m in autumn, and 10–40 m in winter (Figure 2). The order of mean SST > SBT was observed in spring, summer, and autumn; the mean SST and SBT values were similar in winter (Table 1). The lowest SBT values were 10–11 °C in spring and summer (Table 1). The greatest abundance occurred at SBT 14–15 °C in spring, 19 °C in summer, 15–20 °C in autumn, and 10–12 °C in winter (Figure 3). In addition, mean SSS and SBS were similar in autumn, winter, and spring; the order of mean SBS > SSS was observed in summer (Table 1). The greatest abundance occurred at SBS 32–33 in spring, 32 in summer, 32–35 in autumn, and 31–32 in winter (Figure 3).

According to Figure 3, when AIW <0.1 g ind^–1^, SBT was 21 °C and SBS was 34 in autumn; when AIW <0.5 g ind^–1^, SBT values were 12–16 °C and SBS values were 32–34 in winter to spring. When AIW <1 g ind^–1^, SBT values were 10–18 °C in spring, 23–27 °C in summer, and 10–19 °C in winter, and SBS values were 30–34 in spring, 34–35 in summer, and 31–35 in winter. When AIW >1 g ind^–1^, SBT values were 18–20 °C in spring, 11–20 °C in summer, and 15–22 °C in autumn; the SBS values were 33–35 in spring, 32–33 in summer, and 32–35 in autumn.

### 3.2. Spatial Variations in Depth, Water Temperature, and Salinity

In terms of depth, the fishing ground rankings (note: bold italicized words indicate the fishing ground of intensive resource density in each season) were East China Sea (30–100 m) > *Haizhou Bay and Dasha* (20–70 m) > Lvsi (10–30 m) in spring; East China Sea (70–100 m) > *the southern Yellow Sea* (30–40 m) in summer; Mindong (100–120 m) > *Yuwai and Wentai* (80–110 m) > Zhouwai (70–100 m) > Yushan (60–90 m) > Zhoushan (30–60 m) > ***Haizhou Bay* (10–50 m)** in autumn; Yushan (60–130 m) > Zhoushan (60–100 m) > Dasha and Zhouwai (40–70 m) > *Haizhou Bay, Lvsi, and Yangtze River mouth* (20–60 m) in winter (Table 1).

In terms of SST, the fishing ground rankings were as follows: East China Sea (Yangtze River mouth–Zhoushan–Yushan–Wentai–Mindong) (15–25 °C) > *Haizhou Bay and Lvsi* (14–16 °C) > Dasha (13–15 °C) in spring; the northern East China Sea (28–30 °C) > the southern East China Sea (26–29 °C) > ***the southern Yellow Sea* (24–26 °C)** in summer; Mindong (24–25 °C) > Wentai (23–24 °C) > ***Yuwai* (22–24 °C)** > Yushan and Zhouwai (22–23 °C) > Zhoushan (20–22 °C) > ***Haizhou Bay* (18–19 °C)** in autumn; and in terms of SST and SBT, Zhoushan and Yushan (15–19 °C) > Zhouwai (14–17 °C) > Yangtze River mouth and Dasha (12–14 °C) > ***Haizhou Bay and Lvsi* (9–12 °C)** in winter (Table 1).

In terms of SBT, the rankngs were as follows: East China Sea (15–20 °C) > ***Haizhou Bay* (10–15 °C)** > ***Lvsi* (14–15 °C)** > Dasha (12–13 °C) in spring; the southern East China Sea (18–27 °C) > the northern East China Sea (19–24 °C) > ***the southern Yellow Sea* (11–19 °C)** in summer; and Zhouwai (18–23 °C) > Yushan (21–22 °C) > Zhoushan (20–22 °C) > Wentai (19–22 °C) > ***Yuwai* (18–21 °C)** > Mindong (17–18 °C) > ***Haizhou Bay* (15–18 °C)** in autumn (Table 1).

In terms of SSS, the fishing ground rankings were as follows: East China Sea (Yangtze River mouth–Zhoushan–Yushan–Wentai–Mindong) (30–35) > Dasha (32–33) > *Haizhou Bay* (31–33) > ***Lvsi* (31–32.5)** in spring; the southern East China Sea (33–34) > the northern East China Sea (29–34) > ***the southern Yellow Sea* (31–32)** in summer; Mindong (34–35) > *Zhouwai, Yushan, Yuwai, and Wentai* (33–34) > Zhoushan (32–34) > ***Haizhou Bay* (31–32)** in autumn; and Yushan (34–35) > Zhoushan and Zhouwai (33–34) > Yangtze River mouth (32–34) > Lvsi and Dasha (32–33) > ***Haizhou Bay* (31–33)** in winter (Table 1).

In terms of SBS, the fishing ground rankings were as follows: East China Sea (30–35) > Dasha (32–34) > *Haizhou Bay and Lvsi* (31–33) in spring; East China Sea (34–35) > ***the southern Yellow Sea* (32–33)** in summer; Mindong, Wentai, Yushan, and Zhouwai (34–35) > *Yuwai (33–35)* > Zhoushan (32–34) > ***Haizhou Bay* (31–32)** in autumn; and Yushan (34–35) > Zhouwai (33–35) > Zhoushan (33–34) > ***Yangtze River mouth, Dasha, Lvsi, and Haizhou Bay* (32–33)** in winter (Table 1).

### 3.3. Seasonal Variations in CPUE_n_, CPUE_w_, and AIW

These data were collected in autumn (November 2018: 11,511.31 g∙h^−1^ [18.89%] of the total catch per unit effort by weight [CPUE_w_] and 12,357.59 [17.84%] ind∙h^−1^ of the total catch per unit effort by number [CPUE_n_]), winter (January 2019: 28,825.53 [47.3%] g∙h^−1^ of the total CPUE_w_ and 34,387.3 ind∙h^−1^ [49.64%] of the total CPUE_n_), spring (April–May 2019: 19,521.3 g∙h^−1^ [32.03%] of the total CPUE_w_ and 22,225.57 ind∙h^−1^ [32.08%] of the total CPUE_n_), and summer (August–September 2019: 1085.41 g∙h^−1^ [1.78%] of the total CPUE_w_ and 306.06 ind∙h^−1^ [0.44%] of the total CPUE_n_). The annual mean CPUE_w_ and CPUE_n_ were 15,235.89 g∙h^−1^ and 17,319.13 ind∙h^−1^.

The total CPUE_w_ and CPUE_n_ in different seasons were ranked in the following order: winter > spring > autumn > summer, and the mean and upper limit values of CPUE_n_ seasonally ranked in spring > winter > autumn > summer (Table 2). The mean AIW ranking was summer > spring > winter > autumn (Table 2).’

The mean CPUE_w_ and CPUE_n_ rankings for the fishing grounds in spring were as follows: Lvsi (~50%) > Haizhou Bay (~20%) and Dasha (~20%) > East China Sea (Yangtze River mouth, Zhoushan, Yushan, Wentai, and Mindong) (< 10%); Haizhou Bay–Lvsi (90%) > East China Sea (<10%) in summer; Yuwai (60%) > Haizhou Bay (25%) > Zhoushan, Zhouwai, Yushan, Wentai, and Mindong (East China Sea) (<10%) in autumn; and Haizhou Bay (95%) > others (Lvsi, Dasha, Yangtze River mouth, Zhoushan, Zhouwai, and Yushan) in winter (Table 1). The longitudinal rankings for the mean CPUE_w_ and CPUE_n_ were as follows: 121–122.5° E > 123–125° E in spring; 120.5–121.5° E > 122.5–126.5° E in summer; 125–125.5° E > 121.5–122° E > 124–124.5° E and 126–127° E > 123–123.5° E in autumn; and 121–121.5° E >> 122–127° E in winter (Figure 2).

The mean AIW rankings for the fishing grounds were as follows: East China Sea > Dasha > Haizhou Bay and Lvsi in spring; Haizhou Bay–Lvsi (the southern Yellow Sea) > Yushan–Wentai (the southern East China Sea) > Yangtze River estuary–Zhoushan (the northern East China Sea) in summer; Mindong > Yushan > Wentai and Haizhou Bay > Zhoushan > Zhouwai and Yuwai in autumn; and Yushan > Yangtze River mouth, Zhoushan, and Zhouwai > Haizhou Bay and Lvsi > Dasha in winter (Figure 2 and Table 1). The longitudinal ranking of AIW was as follows: 123–125° E > 121–122.5° E in spring; 120.5–123.5° E > 124–126.5° E in summer; 121.5–124.5° E > 125–127° E in autumn; and 126–127° E > 121–124.5° E > 125–125.5° E in winter (Figure 2 and Table 1).

## 4. Discussion

Regarding the spawning period, M. dalei spawns from May to August in Zhejiang, China [5]. In Tosa Bay, Japan, M. dalei spawns from April to November [8], and in Korea, the same species spawns from early July to late August [6]. This indicates that the maturation and spawning period of penaeid shrimps is shorter in cooler waters than in tropical marine environments [9]. In Japanese coastal waters, low ambient temperatures represent a key limiting factor that inhibits the maturation and spawning of penaeid shrimps [8]; indeed, mature females have been documented to occur exclusively during July and August in Suo-nada and Sendai Bay [10]. In the Iyo-Nada area off the Yamaguchi Prefecture in Japan, the majority spawned at SBT ~18 °C in late June to early October, and the larvae shifted to benthic life at 18 days after spawning [11]. On the west coast of Korea, M. dalei produced one cohort per year, and their mean gonadosomatic index reached the maximum value between July and August [6].

**Figure 4:**
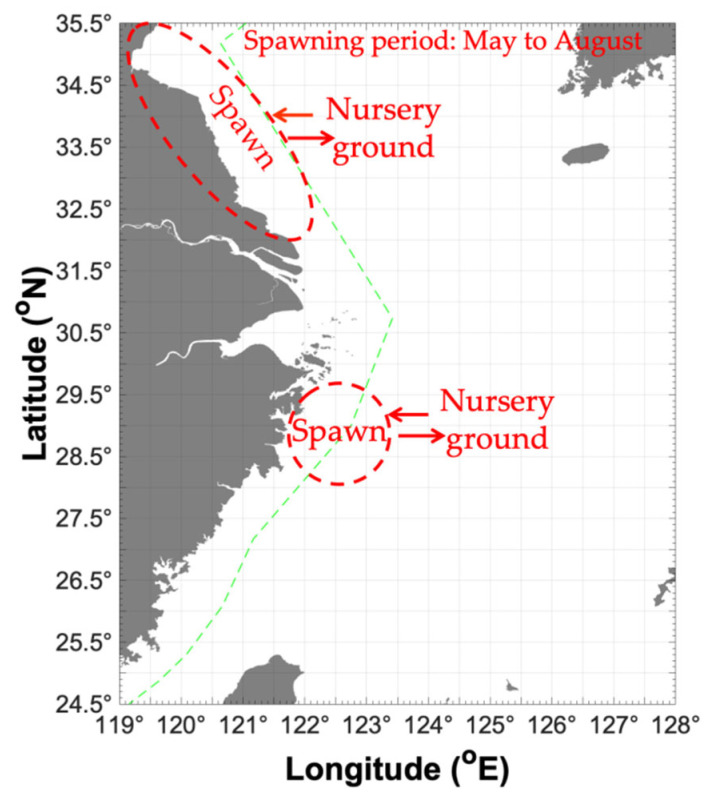
Map of possible migration patterns for *Metapenaeopsis dalei* across seasons in China.

In our study, in spring (May), the growing recruitment cohort in the previous year mainly concentrated on Haizhou Bay, and part of it in Lvsi, at a depth of 20–70 m, SST of 14–16 °C, SBT of 10–15 °C (the majority in 14–15 °C), SSS of 31–32, and SBS of 31–33 (Table 1 and Figure 4). The individual size in the East China Sea was larger than that in the southern Yellow Sea (Table 1). Smaller individuals preferred SBT 12–16 °C SBS 32–34, whereas larger individuals preferred SBT 18–20 °C SBS 33–35. In Japan, they are concentrated at a depth of 10–50 m (Figure 3) [7].

In summer (August), which was the end of the breeding period, the parent cohort mainly concentrated at Haizhou Bay–Lvsi, at a depth of 30–40 m, SST of 24–26 °C, SBT of 11–19 °C (the majority at 19 °C), SSS of 31–32, and SBS of 32–33 (Table 1 and Figure 4). Smaller individuals were found at SBT 23–27 °C and SBS 34–35, and larger individuals were found at 11–20 °C and 32–33 (Figure 3), indicating that the recruitment occurred under high water temperature and salinity, and the surviving parent cohort was concentrated in the water area with a wider range of water temperature and low salinity. The individual size in the southern Yellow Sea was larger than that in the East China Sea (Table 1). In the fishing grounds of Zhejiang, *M. dalei* was mainly found at 20–60 m, especially at the depth of 40–60 m; *Metapenaeopsis dalei* was concentrated in the Zhoushan fishing ground and captured in summer with larger individual size from May to August [5].

In autumn (November), the recruitment was mainly concentrated at Haizhou Bay and Yuwai; the environmental factors in Haizhou Bay were a depth 10–50 m, SST 18–19 °C, SBT 15–18 °C, SSS 31–32, and SBS 31–32, whereas the environmental factors in Yuwai were 80–110 m, SST 22–24 °C, SBT 18–21 °C, SSS 33–34, and SBS 33–35 (Table 1 and Figure 4). The juveniles were found at SBT 21 °C and SBS 34, and larger recruitment cohorts were found at SBT 15–22 °C and SBS 32–35 (Figure 3). Overall, the study area in autumn mainly contained the basic recruitment population (Table 2).

In winter (January), the growing recruitment in this year concentrated on Haizhou Bay, where the environmental factors were a depth of 20–60 m, water temperature 9–12 °C (the majority in 10–12 °C), SSS 31–33, and SBS 32–33 (Table 1 and Figure 4). The mean cohort size decreased from the East China Sea to the Yellow Sea, indicating that a higher water temperature can benefit the growth of *M. dalei* (Table 1).

In the southern Yellow and East China Seas, the spawning ground of *M. dalei* mainly included the Haizhou Bay–Lvsi fishing grounds, with a possible spawning period of May to August, and the parent cohort disappeared after releasing the nauplii [5]. The number of *M. dalei* was the highest in May and the lowest at the end of August (Table 2). The mean individual size of *M. dalei* was the largest in May and August, and the smallest in November (Table 2). In spring, the majority were concentrated in the inshore water area of 121–123° E, comprising smaller individuals in this area, and larger individuals were found in waters to the east of 123° E. In summer, larger individuals were found at 120.5–121.5° E, indicating a possible breeding period in the inshore water area [5] (Figure 4). In autumn, recruitment cohorts were found in the study area, and as the water temperature decreased, recruitment might have migrated from the coastal area to the offshore water area for nursery and feeding. In winter, the cohorts were concentrated in the coastal water area for the nursery, with larger individuals in the offshore area and smaller individuals in the inshore area (Figure 4). During the breeding season, diluted freshwater exerted a strong influence on the survival rate and release of nauplii, at the lowest SBT of 10–11 °C and the depth range of 10–40 m [5] (Table 1).

Similarly, in Suo-Nada, located in the Seto Inland Sea of Japan, Metapenaeopsis dalei is known to consist of two distinct cohorts: early and late. Immediately after settlement, juvenile individuals exhibit a modal carapace length of 1.7 mm [7]. The early cohort is hypothesized to undertake offshore migration in response to declining water temperatures [7]. Both cohorts are thought to spawn at a depth of approximately 25 m during June [7]. The maximum growth rate was estimated at ~1.5 mm per month from April to June, whereas growth ceases entirely when water temperatures drop below 13 °C [7]. Extremely low temperatures are also regarded as a key driver of high mortality rates in the late cohort [7].

Since the 1990s, fisheries management measures implemented in China have encompassed vessel buyback schemes, fishermen relocation and resettlement programs, fishing moratoriums (closed seasons and areas), a total allowable catch (TAC) system, and zero-growth/negative-growth targets—all aimed at curbing the depletion of fishery resources [12]. The coastal environment in the Haizhou-Lvsi fishing grounds, sea-bottom temperature, and freshwater input are vital to the survival rate of juveniles and spawning. Thus, continuous monitoring of the sea bottom temperature in the spawning ground, protective actions in the key spawning period from June to August, and evaluation of the effect of artificial construction and engineering in the spawning ground are essential.

## 5. Conclusions

The key findings from our investigation into the seasonal distribution and migration route of *M. dalei* are as follows: (1) although *M. dalei* was found in the depth range of 10–130 m, it was concentrated at the depth of 10–40 m; (2) in Japan, the distribution pattern of *M. dalei* was described in 33–41° N, while we described the seasonal distribution pattern in 26–35° N of China’s Seas, consuming Haizhou Bay–Lvsi and Zhoushan–Yushan fishing grounds as important nursery and spawning grounds for *M. dalei*; (3) further, the lower water temperature in North China may be able to elongate the breeding period of *M. dalei*, but higher water temperature may benefit the growth of *M. dalei*.

## Figures and Tables

**Table 1:** Summary statistics of catch metrics across seasons, environmental conditions, and fishing grounds (refer to Figure 1). Mean and total values of weight-based catch per unit effort (CPUE_w_, g h^−1^), percentage contribution of CPUE_w_, numerical catch per unit effort (CPUE_n_, ind h^−1^), percentage contribution of CPUE_n_, and average individual weight (AIW, g ind^−1^), stratified by season, environmental parameters (sea surface temperature (SST), sea surface salinity (SSS), sea bottom temperature (SBT), sea bottom salinity (SBS), and water depth), and fishing ground. Region I in the table corresponds to the combined fishing grounds of the Yangtze River Estuary, Zhoushan, Yushan, Wentai, and Mindong.

Survey Area	Mean Value					Total Value				Environmental Factors				
	CPUE_w_	CPUE_w_%	CPUE_n_	CPUE_n_%	AIW	CPUE_w_	CPUE_w_%	CPUE_n_	CPUE_n_%	SST	SSS	SBT	SBS	Depth
Spring														
Haizhou Bay	199.8	21.9%	288.9	23.2%	0.69	16,554.5	84.8%	18,357.9	82.6%	13.7–15.1	30.8–32.8	10.1–14.6	30.8–33.1	20–73
Lvsi	466.7	51.1%	655.8	52.8%	0.63	1867	9.6%	2623.4	11.8%	14.2–15.7	30.9–32.4	14.4–15.1	31.4–32.3	13–25
Dasha	188.4	20.6%	273.4	22%	0.83	753.5	3.9%	1093.8	4.9%	12.9–14.6	31.9–33.2	11.8–12.8	32.2–33.7	36–65
Region I	57.7	6.3%	25.1	2%	2.88	346.3	1.8%	150.7	0.7%	15–24.6	30.6–34.3	15–19.7	30.6–34.7	36–98
Summer														
Haizhou Bay–Lvsi	255.3	93.2%	67.7	86.8%	3.95	1021	94.1%	271	88.5%	24.6–25.5	31.1–31.7	11–19.3	32.2–33	30–40
Yangtze River mouth–Zhoushan	9.8	3.6%	6.1	7.8%	1.71	29.4	2.7%	18.2	6%	28.5–29.6	28.9–33.5	19.4–23.6	34.2–34.3	70–96
Yushan–Wentai	8.7	3.2%	4.2	5.4%	1.92	35	3.2%	16.9	5.5%	26.4–28.6	33.7–34	17.9–26.6	34–35	70–101
Autumn														
Haizhou Bay	595.8	23.1%	589.3	21.1%	0.91	1787.4	15.53%	1768	14.3%	18–19.1	31.8–31.9	15.3–18.1	31.7–32	14–48
Zhoushan	30.15	1.2%	104.1	3.7%	0.7	120.6	1%	416.3	3.4%	20–21.6	32.2–33.6	20–21.6	32.3–33.9	35–60
Zhouwai	126.2	4.9%	191.5	6.9%	0.66	378.61	3.3%	574.6	4.7%	22.6–23.1	34	18.6–22.6	34.1–34.6	73–100
Yushan	101.6	3.9%	104.6	3.8%	1.03	507.91	4.4%	522.8	4.2%	21.7–22.8	33.3–34.1	21.1–21.7	34.1–34.4	66–85
Yuwai	1506.2	58.3%	1578.8	56.6%	0.61	7530.8	65.4%	7893.9	63.9%	21.9–23.4	33.3–34.3	18.5–20.6	33.5–34.4	82–107
Wentai	172.7	6.7%	174.7	6.3%	0.94	1036	9%	1048	8.5%	23–24	33.4–34.3	19.8–21.6	34–35	83–100
Mindong	50	1.9%	44.7	1.6%	1.19	150	1.3%	134	1.1%	23.7–24.8	34.3–34.4	17.6–18.3	34–35	96–115
Winter														
Haizhou Bay	4513	94.9%	5473.8	96.1%	0.82	27,078.1	93.9%	32,842.7	95.5%	9.8–11.8	31.5–32.3	10–12	31.8–32.4	17–62
Lvsi	19.5	0.4%	21.7	0.4%	0.85	58.5	0.2%	65	0.2%	9.1–11.9	32.1–32.5	9.3–11.9	32–32.4	19–40
Dasha	9.4	0.2%	13.2	0.2%	0.75	37.7	0.1%	52.7	0.2%	12–13.2	32.5–33.1	12.3–13.2	32.7–33	37–65
Yangtze River mouth	24.2	0.5%	24.7	0.4%	1	169.2	0.6%	172.8	0.5%	11.7–13.5	32.4–33.4	11.9–13.7	32.6–33.4	35–48
Zhoushan	104.3	2.2%	93.9	1.7%	1	834.2	2.9%	751.1	2.2%	14.7–18.3	33.2–34.1	14.8–18.3	33.2–34.1	58–100
Zhouwai	29	0.6%	34.7	0.6%	0.96	232.2	0.8%	277.5	0.8%	14.3–16.6	33.5–34.3	14.4–16.6	33.5–34.4	41–66
Yushan	59.4	1.3%	32.2	0.6%	1.4	415.7	1.4%	225.6	0.7%	15–19.8	33.9–34.5	15.2–18.4	34–35	60–126

**Table 2:** Seasonal datasets on weight-based catch per unit effort (CPUE_w_, g h^−1^), numerical catch per unit effort (CPUE_n_, ind h^−1^), and average individual weight (AIW, g ind^−1^), collected from autumn 2018 through summer 2019.

Variable	Spring	Summer	Autumn	Winter
Mean CPUE_w_ at collection stations	976.1	98.7	396.9	670.4
Value range of CPUE_w_	0.93–16,311.6	1.66–791.6	0.86–7411.2	1.2–10,771
Mean CPUE_n_ at collection stations	1111.28	27.82	426.1	799.7
Value range of CPUE_n_	1–18,021	1–212	1.07–7680	2–15,168
Mean AIW	1.47	2.6	0.86	0.99
Value range of AIW	0.35–5.4	0.6–4.7	0.06–1.54	0.3–2.7

## Data Availability

The original contributions presented in this study are included in the article. Further inquiries can be directed to the corresponding authors.
